# Repeated verum but not placebo acupuncture normalizes connectivity in brain regions dysregulated in chronic pain

**DOI:** 10.1016/j.nicl.2015.09.012

**Published:** 2015-09-25

**Authors:** Natalia Egorova, Randy L. Gollub, Jian Kong

**Affiliations:** Department of Psychiatry, Massachusetts General Hospital/Harvard Medical School, Charlestown, USA

**Keywords:** Acupuncture, Resting state fMRI, Chronic pain, Osteoarthritis

## Abstract

Acupuncture, an ancient East Asian therapy, is aimed at rectifying the imbalance within the body caused by disease. Studies evaluating the efficacy of acupuncture with neuroimaging tend to concentrate on brain regions within the pain matrix, associated with acute pain. We, however, focused on the effect of repeated acupuncture treatment specifically on brain regions known to support functions dysregulated in chronic pain disorders. Transition to chronic pain is associated with increased attention to pain, emotional rumination, nociceptive memory and avoidance learning, resulting in brain connectivity changes, specifically affecting the periaqueductal gray (PAG), medial frontal cortex (MFC) and bilateral hippocampus (Hpc). We demonstrate that the PAG–MFC and PAG–Hpc connectivity in patients with chronic pain due to knee osteoarthritis indeed correlates with clinical severity scores and further show that verum acupuncture-induced improvement in pain scores (compared to sham) is related to the modulation of PAG–MFC and PAG–Hpc connectivity in the predicted direction. This study shows that repeated verum acupuncture might act by restoring the balance in the connectivity of the key pain brain regions, altering pain-related attention and memory.

## Highlights

• Increased PAG–hippocampus connectivity correlates with worse pain in knee osteoarthritis.
• Reduced PAG–medial frontal connectivity is associated with worse pain during sport.
• Verum acupuncture decreases PAG–hippocampus connectivity improving pain scores.
• Sham acupuncture decreases PAG–medial frontal connectivity with no pain improvement.

## 1 Introduction

In recent years, acupuncture has gained popularity in Western medicine in part due to successful treatment of chronic pain (White, 2009). Despite its increasingly widespread use by the general population, clinical trials evaluating the efficacy of acupuncture report inconsistent results. Often they show the general advantage of acupuncture over other physical interventions (Corbett et al., 2013; Manyanga et al., 2014; Vickers and Linde, 2014). The lack of understanding of the underlying mechanisms of acupuncture and reported cases of a failure to elicit greater clinical improvement compared to sham acupuncture (Hinman et al., 2014; Kong et al., 2013; Langevin et al., 2011), however, have hampered incorporation of this treatment modality into mainstream medical practice.

Functional neuroimaging investigation into the mechanisms of acupuncture shows that acupuncture needle stimulation is associated with fMRI signal increase in the insula, thalamus, and primary and secondary somatosensory cortices, signal decrease in the caudate, posterior cingulate cortex, thalamus and parahippocampus, as well as both signal increase and decrease in the medial prefrontal cortex (mPFC), anterior cingulate, amygdala and cerebellum (Chae et al., 2013). In addition, connectivity between these pain regions and the periaqueductal gray (PAG) is modulated during acupuncture (Zyloney et al., 2010) and differentiates verum (genuine) and sham treatments. However, to date neuroimaging studies focused on either the brain regions responding to acute pain sensation and/or measured resting state changes during or immediately after an acupuncture session. Long-term treatment effects and changes in brain regions associated with chronic pain remain unknown. In parallel, substantial effort has gone into identifying the brain regions and networks that show significant changes during the transition from acute to chronic pain (Apkarian et al., 2011; Hashmi et al., 2013), associated, inter alia, with long-term pain learning (Mansour et al., 2014). Most often the medial frontal cortex (MFC) (Baliki et al., 2011, 2014; Cauda et al., 2014; Mutso et al., 2013) and bilateral hippocampus (Hpc) (Gondo et al., 2012; Lang et al., 2009; Mutso et al., 2013; Ploghaus et al., 2001; Vachon-Presseau et al., 2013) are implicated. These brain regions appear to be the hubs involved in pain perception (Gondo et al., 2012; Salomons et al., 2007), placebo analgesia (Eippert et al., 2009; Wager et al., 2007), pain-related memory and emotion (Lang et al., 2009; Ploghaus et al., 2001), and as such control pain perception and modulation.

Of special relevance is the connectivity between the MFC, Hpc and the PAG. While both MFC and Hpc are directly involved in any type of learning and memory consolidation and retrieval (Carter et al., 2006; van Kesteren et al., 2010), the PAG is specifically implicated in aversive learning and prediction error generation, which is crucial for forming long-term pain behavior and nociceptive memory (Roy et al., 2014). If pain behavior can be unlearned by altering the aberrant brain connectivity patterns of the PAG in its capacity as a pain-learning brain region, this could potentially stop or even reverse the transition to chronic pain. This led us to investigate whether acupuncture, known to work through the descending pain modulation system (Kong et al., 2010), including the PAG, could be instrumental in normalizing chronic pain brain circuitry.

In this study we examined the relationship between chronic knee osteoarthritis symptoms (measured with the Knee injury and Osteoarthritis Outcome Score (KOOS) pain and sport subscales) and resting state connectivity between PAG–MFC and PAG–Hpc, as both MFC and Hpc are crucial for pain learning, attention and memory. We hypothesized that lower PAG–Hpc connectivity (associated with less attention to pain, anxiety and nociceptive memory) would correlate with better pain and sport scores; in contrast, lower PAG–MFC connectivity (indicating increased attention to pain, low analgesia expectation, higher pain avoidance) would correlate with increased pain and sport scores. We also hypothesized that connectivity in PAG–Hpc and PAG–MFC would be improved by repeated verum acupuncture (compared to sham).

## 2 Materials and methods

The full details of subject enrollment and inclusion, study design, acupuncture procedures and fMRI acquisition are reported elsewhere (Chen et al., 2014; Spaeth et al., 2013). Here we provide a brief description of the methods and focus on the procedures and analysis details relevant for this study.

### 2.1 Subjects

Forty-four knee osteoarthritis patients (19 females) were enrolled in the study, of which 30 (13 females, aged 57.5 ± 8.3, mean ± SD) completed all experimental stages. The Institutional Review Board of the Massachusetts General Hospital approved all study procedures. The subjects provided informed consent at the beginning of the study and were debriefed at the end of the study.

### 2.2 Experimental design

Subjects in this study had either unilateral or bilateral knee pain, however, they all received acupuncture treatment on either right or left knee (whichever presented with more severe pain). Subsequent treatment and pain assessment only focused on one knee. Subjects were randomized into one of the three groups: 1) verum acupuncture at 6 acupoints, 2) verum acupuncture at 2 acupoints, or 3) sham acupuncture (6 acupoints with Streitberger placebo needles), keeping knee condition (bilateral/unilateral pain predominantly in right/left knee) balanced across groups. After the initial screening, each subject received a total of 6 acupuncture sessions within one month (twice a week during weeks 1–2 and once a week during weeks 3–4). Treatments 1, 3, and 6 were conducted with the patient lying in a 3 Tesla MRI scanner. Treatments 2, 4 and 5 took place in a behavioral testing room.

Verum acupuncture was performed on ST35 and Xiyan points for the low-dose verum groups and additionally at GB34, SP9, GB39 and SP6 for the high-dose group. Both ST35 and Xiyan points are located near the knee. They are typically used in knee pain treatment and have been previously used in osteoarthritis clinical trials (Berman et al., 2004; Cheng, 1987; Scharf et al., 2006; Stux, 1997; Witt et al., 2005). Placebo acupuncture was performed in 6 spots on the lower leg, where no meridians pass through. All subjects received acupuncture treatment on the ipsi-lateral side (i.e., on the painful knee); subjects receiving acupuncture at 6 points had acupuncture performed on both legs, but never uniquely on the contralateral side (Yi et al., 2011).

Each acupuncture session lasted approximately 25 min and was the same for all subjects, regardless of the group assignment. For the purposes of this analysis, subjects in the two verum acupuncture groups were combined, as the two treatment types evoked similar *deqi* sensation, had the same stimulation time and treatment effect, as indicated by KOOS (Spaeth et al., 2013). Compared to verum acupuncture, in which the needle penetrates the skin, sham acupuncture was performed with non-penetrating Streitberger needles (the needle retracts up into the shaft when pressed against the skin). Subjects were blind to the site of needle insertion and to whether the treatment was verum or sham. For the duration of the treatment, subjects agreed not to receive any other medical intervention or medication for their knee osteoarthritis.

Treatment clinical outcomes were measured using the KOOS (Roos et al., 1998), comprising 5 subscales: 1) pain, 2) other symptoms, 3) function in daily living, 4) function in sport and recreation, and 5) knee-related quality of life. Each subscale is measured from 0–100, with a greater score signaling improvement. The KOOS was administered to all patients at baseline (up to one week before treatment 1) and after the final treatment (treatment 6). The scale requires patients to assess their state during the preceding week. Based on our hypotheses and previous results showing significant differences between sham and verum acupuncture (Chen et al., 2014), we only focused on 2 subscales: pain and sport, the latter representing pain ratings during increased physical activity; the other subscales were not analyzed.

### 2.3 fMRI acquisition and pre-processing

**Fig. 1:**
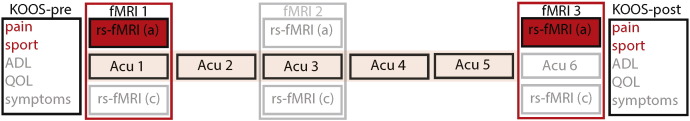
Study design. Elements of the study design marked in red are relevant for the current report. Elements of the original design marked in gray were not included in the analysis (see ref). Baseline KOOS-pre pain and sport values were used to investigate a correlation between baseline resting state connectivity of PAG–mPFC and PAG–Hpc. The post-acupuncture resting state took place following 5 acupuncture sessions (marked in pink). KOOS-post pain and sport measurements were collected after 6 acupuncture sessions and are mentioned in this study only to show pain and sport KOOS improvement in the verum compared to sham group but are not analyzed in relation to the reported resting state changes. The main analysis is focused on the comparison of the baseline and post-acupuncture resting state in the two acupuncture groups and their relation to baseline KOOS-pre scores.

Each subject underwent 3 fMRI scanning sessions. Each scanning session included a 6-minute resting state scan (a), two functional scans during acupuncture (25 min) (b), as well as another 6-minute resting state scan (c), see Fig. 1. We here focus only on the resting state scans (a) before acupuncture treatment during fMRI session 1 and session 3. Resting state session 1 (henceforth ‘pre’-acupuncture resting state) was performed before the beginning of repeated acupuncture treatment and within about a week of the measurement of the baseline KOOS (KOOS-pre). Resting state session 3 (henceforth ‘post’-acupuncture resting state) was performed approximately 25 days later following 5 acupuncture treatments but before the final (6th) acupuncture treatment. For the resting state scan subjects were required to keep their eyes open. The final KOOS (KOOS-post) was measured on the same day but after the 6th acupuncture treatment was performed, see Fig. 1.

Scanning was performed using a 3-axis gradient head coil in a 3 Tesla Siemens MRI scanner with echo planar imaging (EPI). Structural scans were acquired using a magnetization prepared rapid gradient echo (MPRAGE) sequence with TR = 2200 ms, TE = 9.8 ms, flip angle of 7°, field of view of 230 mm^2^, and slice thickness of 1.2 mm. For the resting state analysis, 47 slices were acquired with the following parameters: TR = 3000 ms, TE = 30 ms, flip angle of 85°, the field of view of 216 mm^2^, and 3 mm ∗ 3 mm ∗ 3 mm in-plane spatial resolution.

The preprocessing of resting state images was done using SPM 8 software (http://www.fil.ion.ucl.ac.uk/spm) implemented in a MATLAB suite (Mathworks, Inc., Natick, Massachusetts). It included slice time correction, head motion correction, co-registration to subjects' structural images, segmentation, normalization, linear detrending and smoothing (FWHM = 6 mm).

Functional connectivity analysis was carried out with the CONN toolbox (www.nitrc.org/projects/conn) (Whitfield-Gabrieli and Nieto-Castanon, 2012). Timecourses from the components associated with white matter and cerebrospinal fluid (CSF) were regressed out of whole-brain gray matter activity, 12 motion regressors (6 realignment parameters and first derivatives) were used to control for correlations during movement. Data were filtered between 0.008 and 0.09 Hz, global brain signal was not subtracted.

Functional connectivity analysis was performed using an ROI-to-ROI approach. PAG was used as a seed ROI. As PAG mask is not available in the Harvard–Oxford atlas implemented in the CONN toolbox by default, a bilateral PAG ROI mask was created using Mango software (http://rii.uthscsa.edu/mango/) based on several of the previous coordinates (± 6, − 30, − 14 (Leknes et al., 2013); ± 4, − 26, − 14 (Kong et al., 2010; Yu et al., 2014); ± 2, − 26, − 10 (Amanzio et al., 2013; Derbyshire and Osborn, 2009)) reported in representative pain/analgesia studies and meta-analyses. We created partially overlapping spheres (3-mm radius) around the given coordinates to form one mask (volume 808 mm^3^) and used it for further analysis. We chose to create this functionally rather than an anatomically defined mask, as PAG is involved in a number of functions not related to pain. This functionally defined mask covers the coordinates reported for pain, acupuncture and placebo, as identified in a recent meta-analysis (Linnman et al., 2012b), as well as a number of other studies (Kucyi et al., 2013; Zyloney et al., 2010). Connectivity of PAG seed with the anatomically defined bilateral Hpc (combined volume 38,189 mm^3^) and MFC (volume 30,833 mm^3^) (based on the Harvard–Oxford cortical and subcortical structural atlases, implemented in CONN) was examined.

In the first-level analysis, we produced a correlation map for each subject by extracting the BOLD time course from the PAG seed ROI and computing Pearson's correlation coefficients between the time course in the PAG and the MFC and Hpc. Connectivity values between the PAG seed and the Hpc and MFC ROIs for the pre-acupuncture and post-acupuncture sessions were extracted for each subject.

First, we used the PAG–MFC and PAG–Hpc connectivity values for all 30 patients and correlated them with the pre-acupuncture KOOS pain and KOOS sport scores, in order to determine whether these behavioral measures correlated with the PAG–MFC and PAG–Hpc connectivity at baseline. Importantly, identifying the direction of the correlation (positive or negative) with the clinical outcome would allow us to understand how the connectivity needs to change (decrease or increase) to lead to the improvement in KOOS. As the differences in age and gender have been previously reported to influence MFC, PAG and Hpc connectivity (Hashmi et al., 2014; Hong et al., 2013; Linnman et al., 2012a), we used partial Pearson correlations (controlling for age and gender) to explore the relation of PAG–MFC to pain and sport KOOS. The two subscales were chosen based on a previous study that suggested that acupuncture can provide greater pain relief and improvement in function in patients with osteoarthritis of the knee (Chen et al., 2014); as well as based on our previous study (using the same dataset) showing that verum acupuncture significantly improves KOOS pain and sport scores compared with sham treatment (Spaeth et al., 2013).

After that we performed a second-level connectivity analysis, with a between group factor *acupuncture group* (verum minus sham) and a within subject factor *treatment* (‘post’ — repeated acupuncture treatment, i.e. post 5 acupuncture sessions minus ‘pre’ — acupuncture treatment) to identify the effect of verum vs. sham acupuncture post-treatment compared to pre-treatment on the PAG–MFC and PAG–Hpc connectivity. We included age and gender as covariates. Planned comparisons — between-session differences in each of the acupuncture groups were performed using the CONN toolbox to understand the nature of the change. For all analyses the threshold of p < 0.05 FDR-corrected was used.

In addition, head motion analysis was performed using the artifact detection toolbox (ART) (https://www.nitrc.org/projects/artifact_detect/). Timepoints were marked as outliers if global signal exceeded three standard deviations of the mean and if movement exceeded 0.5 mm of scan-to-scan deviation. As head motion can affect connectivity measures (Power et al., 2012), we examined whether there was a difference between the groups in the number of outliers in each session, using 2-sample t-tests (2-tailed). As we primarily used the contrast between sessions (‘post’ minus ‘pre’) throughout all analyses, we also computed a difference between the number of outliers between the sessions and compared it between the verum and sham groups with a 2-sample t-test (2-tailed).

## 3 Results

### 3.1 Behavioral results

Full behavioral data analysis of the effects of the verum and sham acupuncture treatment is reported elsewhere (Chen et al., 2014; Spaeth et al., 2013). In summary, typical acupuncture sensations, such as perceived soreness, and aching differed significantly between the verum and sham acupuncture groups, suggesting that real acupuncture reliably evoked stronger *deqi* sensations compared to sham, and led to significant improvement (decrease) in knee pain (F_(1,28)_ = 5.596, p = 0.025) and improved function in sport (F_(1,27)_ = 4.252, p = 0.049), as indicated by a repeated measures ANOVA with the Acupuncture group (verum vs. sham) as the between subject factor and Treatment (pre vs. post) as a within subject factor. Post hoc analysis indicated that repeated verum acupuncture produced a significant positive effect on pain (Mean = 12.1, SE = 3.0) and sport clinical outcomes (Mean = 18.4, SE = 5.7), compared to sham. In the sham group almost no difference between KOOS-post minus KOOS-pre was observed for pain (Mean = 0.83, SE = 3.9), and on average slightly negative change was observed for sport (Mean = − 1.5, SE = 6.8).

We here concentrate on the resting state ROI-to-ROI connectivity analysis and its relation to the pre-treatment KOOS pain and sport subscales.

### 3.2 Functional connectivity results

Partial correlation analysis of the baseline PAG–MFC and PAG–Hpc connectivity and baseline KOOS pain and sport subscales controlling for age and gender, revealed a significant negative correlation between the PAG–Hpc connectivity and KOOS pain score r_(N = 30)_ = − 0.40, p = 0.033 (2-tailed), suggesting that increased PAG–Hpc connectivity was associated with worse pain symptoms in patients with osteoarthritis of the knee. It also revealed a significant positive correlation between PAG–MFC connectivity and KOOS sport score r_(N = 30)_ = 0.43, p = 0.021 (2-tailed), showing that higher PAG–MFC connectivity accompanied less difficulty experienced when being active on a higher level (sport and recreational activities). No significant results for the PAG–MFC and KOOS pain or PAG–Hpc and KOOS sport were found.

The analysis of the effect of verum repeated acupuncture treatment (‘post’ minus ‘pre’) compared to sham (verum minus sham) controlling for the between-group differences in age and gender revealed a significant change of the PAG–MFC connectivity, b = 0.22, t_(26)_ = 2.18, p = 0.038, as well as a significant change in the PAG–Hpc connectivity, b = 0.19, t_(26)_ = − 2.34, p = 0.027, both surviving the FDR correction (adjusted p_FDR_ = 0.038). More positive PAG–MFC and more negative PAG–Hpc connectivity following verum, compared to sham acupuncture treatment was observed, consistent with the direction of the correlations of PAG–MFC and PAG–Hpc with KOOS scores at baseline, signaling better brain-connectivity and clinical state.

**Fig. 2:**
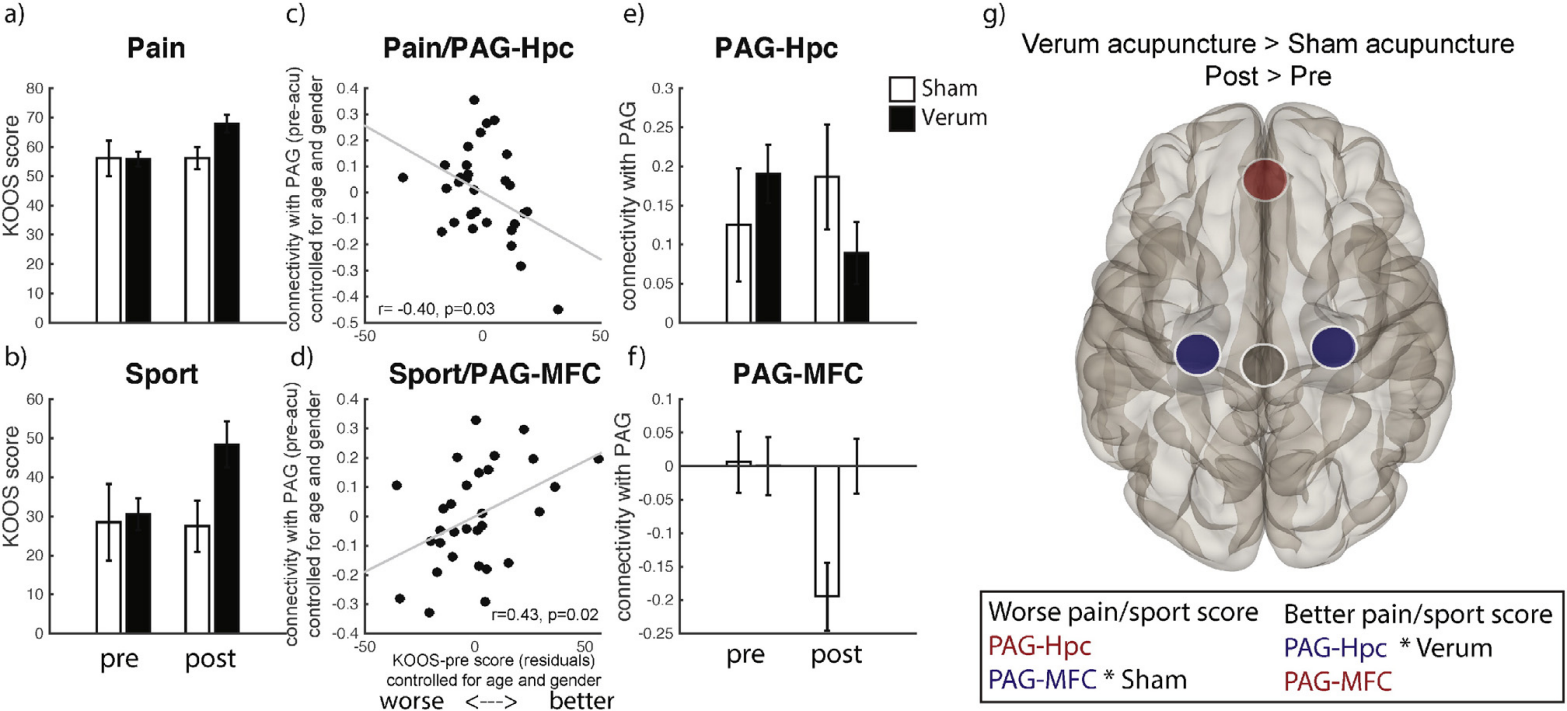
Main results. KOOS scores between verum and sham acupuncture groups pre- and post-acupuncture treatment (a, b). Significant partial correlations between KOOS pain and sport subscales and pre-acupuncture PAG–Hpc and PAG–mPFC connectivity across all patients (c, d). Connectivity values for PAG–Hpc and PAG–mPFC for verum and sham groups pre- and post-acupuncture treatment. Bars represent standard errors (e, f). PAG seed ROI (black) connectivity with Hpc and mPFC for the contrast Verum > Sham, Post > Pre-acupuncture treatment (g). All connectivity values were calculated as the correlation between the time course from the PAG ROI seed and the time course in the mPFC and Hpc ROIs. A decrease in PAG–Hpc connectivity (observed in verum, compared to sham) possibly indicates less attention to pain, anxiety and nociceptive memories; a decrease in PAG–mPFC (observed in sham, compared to verum, resulting in a positive connectivity in the verum > sham contrast as shown here) possibly indicates more attention to pain and a decrease in relief expectation following repeated acupuncture treatment.

In order to understand the nature of the change we plotted the connectivity values for each group (verum and sham) and session (‘pre’ and ‘post’), Fig. 2, and performed planned pairwise comparisons between ‘pre’ and ‘post’ sessions within acupuncture groups, revealing a decrease in PAG–Hpc connectivity in the verum group (b = − 0.12, t_(19)_ = − 3.47, p = 0.002) and a decrease in PAG–MFC in the sham group (b = − 0.2, t_(9)_ = − 2.95, p = 0.016).

No differences in the number of outliers between the verum and sham groups were observed in either of the sessions (‘pre’ — p = 0.23; ‘post’ — p = 0.24), or in the difference between sessions (‘post’ minus ‘pre’ — p = 0.66), suggesting that reported differences between the verum and sham groups in connectivity cannot be explained by head movement.

## 4 Discussion

In this study we investigated the relationship between the connectivity in the brain regions involved in pain learning, attention and memory also implicated in pain chronification, and baseline clinical outcome scores (KOOS pain and sport) in patients with osteoarthritis of the knee. We specifically studied the effect of repeated verum acupuncture (compared to sham) on the change in both connectivity and clinical outcome. We found that PAG–Hpc connectivity was negatively correlated with baseline KOOS pain scores (the lower the connectivity the better the pain score) and that it decreased following repeated verum acupuncture resulting in pain score improvement. We also found that PAG–MFC connectivity was positively correlated with KOOS sport scores (the lower the connectivity the worse the sport score), and that it decreased in subjects who received sham acupuncture, also resulting in minor worsening in the sport score. In contrast, in the verum group maintained PAG–MFC connectivity level was observed together with increased sport scores. Our results suggest that repeated verum acupuncture might reverse chronification in patients by interrupting persistent nociception and reducing attention to pain.

### 4.1 Pain learning and nociceptive memory

Chronic pain has been conceptualized as a type of long-term learning (Apkarian et al., 2011), characterized by accumulation of nociceptive memory (Yi and Zhang, 2011) and inability to extinguish negative emotional associations and anxiety (Mutso et al., 2013; Ploghaus et al., 2001; Vachon-Presseau et al., 2013), in which both Hpc and mPFC are involved (Carter et al., 2006; Lang et al., 2009). In rats, disrupting hippocampal function has been shown to reverse persistent nociception (Ma et al., 2013). In humans, a recently proposed model of aversive prediction error in pain learning has suggested the PAG as the primary hub in the pain learning neural network (Roy et al., 2014) relying on mPFC and Hpc. However, whether the PAG is directly associated with Hpc or their connectivity is mediated through other brain regions, requires further investigation.

In our study, at the time of the first treatment session both the verum and sham groups were naïve to acupuncture, had ongoing pain and had high expectation of relief with treatment as assessed by a pre-treatment rating questionnaire (Spaeth et al., 2013). In terms of connectivity, this stage corresponded to high PAG–Hpc and baseline PAG–mPFC connectivity on average in all subjects (although both positive and negative individual connectivity was observed in the correlation analysis). Following treatment, the connectivity of PAG and Hpc in the verum group decreased. In line with the previous studies, this reduction in connectivity could be the result of updating of nociceptive memory, reducing aversive prediction, leading to lower anticipation and salience of incoming pain.

The *repeated* aspect of the treatment could be crucial for this process of pain-related re-learning, possibly through the immediate pain relief with each verum acupuncture session boosting expectation and introducing positive re-assessment of the pain state. As verum acupuncture was associated with stronger *deqi* sensations (Spaeth et al., 2013), it is possible that it activated the descending pain modulation system, initiating the release of endogenous opioids and inhibiting nociceptive signaling from the periphery (Kaptchuk, 2002; Kong et al., 2009b), maintaining subjects' expectancy and leading to pain improvement as the treatment progressed. In contrast, maintained high PAG–Hpc connectivity level in the sham group could indicate that no prediction or pain behavior update took place, as subjects did not experience pain relief from session to session, apart from the non-specific treatment effects — expectation and ‘placebo’ (Kong et al., 2009a) that decreased over the course of treatment. This speculation is further corroborated by the decrease in the PAG–MFC connectivity in the sham group, possibly reflecting a reduction in relief expectation. Based on these findings, we suggest that pain reduction during repeated verum acupuncture in patients reverses chronification by updating nociceptive memory, while sham acupuncture, relying on expectation alone, fails to maintain this expectation effect in the absence of actual pain relief.

### 4.2 Attention to pain

Another key aspect of pain chronification related to pain learning is increased attention to pain (Durnez and Damme, 2015) and aberrant default mode network (DMN) connectivity in chronic pain conditions, suggesting inability to disengage from attending to painful sensations (Baliki et al., 2014). Distraction from pain and mind-wandering (Kucyi et al., 2013; Tracey et al., 2002; Valet et al., 2004), as well as acupuncture (Li et al., 2014), have been associated with enhanced PAG and increased mPFC activations suggesting that correlated activity between the two regions results in reduced pain sensation. In line with this, we also found a significant positive association between PAG–mPFC connectivity and KOOS sport score at baseline, suggesting that greater PAG–mPFC connectivity is associated with less difficulty engaging in increased physical activity, such as performing sport and recreational activities on a higher level. This subscale reflects increased motivation, reduced pain avoidance and emphasis on overcoming the discomfort, likely by shifting attention away from pain and changing pain processing from fixed negative emotional back to sensory evaluation (Hashmi et al., 2013).

Following acupuncture treatment, subjects in the verum group felt less pain and also demonstrated improvement in sport (increased physical activity), which was coupled with maintained baseline PAG–mPFC connectivity. This pattern of mPFC activation and PAG–mPFC connectivity has been observed in healthy subjects not paying attention to pain, as reported in a previous study (Kucyi et al., 2013), showing baseline DMN activity levels (i.e. no activation) when attention fluctuated away from pain. The same study also reported a decrease in PAG–mPFC connectivity when subjects paid attention to pain (and felt more pain).

Another study on chronic low back pain also linked decreased PAG–mPFC connectivity with worse pain ratings following a pain-inducing maneuver, i.e. increased physical activity similar to that implied in the KOOS sport subscale (Yu et al., 2014). Consistent with these previous findings, we observed decreased PAG–mPFC connectivity in the sham group, possibly representing a sustained focus on pain, which was accompanied by virtually no behavioral pain score improvement and, if anything, a decrease in the sport score. This relationship between pain and attention potentially has implications for pain behavior. The difference in connectivity and behavioral patterns between verum and sham acupuncture in our study suggests a possible difference in coping behavior in the two groups. Subjects treated with verum acupuncture seemed to exhibit more pain tolerance following treatment, engaging in sport and experiencing less pain, possibly by paying less attention to it, while sham acupuncture group subjects likely continued to focus on their pain and use pain avoidance strategies.

One limitation of the study is that we did not collect any behavioral measures of attention or memory function to confirm a direct relationship with the observed connectivity changes. Future research could capitalize on these preliminary findings to further investigate the relationship between attention and memory improvement following acupuncture. Note, however, that we do not claim any specific acupuncture-induced improvement in memory and attention in general, but only discuss attention and memory as they relate to pain, placebo and coping strategies, based on previous literature.

Although the study was initially designed to test differences between low and high dose acupuncture, we did not make the distinction between the two in the connectivity analysis, due to the absence of behavioral differences in the clinical outcome between the two groups. As the differences in the neural responses are likely to be subtle, larger sample size to achieve sufficient power teasing apart behavioral and connectivity differences would be required. However, the more prominent distinction between verum and sham acupuncture reported here, was apparent in both behavioral and connectivity measures with this sample size.

We, therefore, speculate that immediate relief from verum acupuncture as part of a series of treatments could help maintain strong relief expectation, ultimately resetting previously learned pain behavior and leading to less avoidance and attention to pain that characterizes chronic pain states. Our results suggest that measuring resting state PAG–mPFC and PAG–Hpc connectivity could be used to provide an estimate of chronification in patients with chronic pain disorders. In this study a relatively small sample of 30 patients was examined; further validation of PAG–mPFC and PAG–Hpc connectivity as a marker of pain severity and a measure of success of acupuncture treatment is required.

## 5 Conclusion

This study showed how repeated verum acupuncture treatment changes the behavioral outcomes, as well as the connectivity in key brain regions associated with pain learning, memory and chronification. The results suggested that repeated verum acupuncture might normalize patients' attention to pain and reset fixed predictive, motivational and emotional learning, changing patients' pain behavior and reinstating the healthy balance within the mind, body and brain. These results have implications for the assessment of the efficacy of acupuncture treatment with regard to reversal of chronic pain disorders.
